# Bisphenol A Inhibits the Transporter Function of the Blood-Brain Barrier by Directly Interacting with the ABC Transporter Breast Cancer Resistance Protein (BCRP)

**DOI:** 10.3390/ijms22115534

**Published:** 2021-05-24

**Authors:** Elin Engdahl, Maarten D. M. van Schijndel, Dimitrios Voulgaris, Michela Di Criscio, Kerry A. Ramsbottom, Daniel J. Rigden, Anna Herland, Joëlle Rüegg

**Affiliations:** 1 Environmental Toxicology, Department of Organismal Biology, Uppsala University, 75236 Uppsala, Sweden; maarten.van.schijndel95@gmail.com (M.D.M.v.S.); michela.dicriscio@ebc.uu.se (M.D.C.); joelle.ruegg@ebc.uu.se (J.R.); 2 Division of Micro and Nanosystems, Department of Intelligent Systems, KTH Royal Institute of Technology, 11428 Stockholm, Sweden; dvou@kth.se (D.V.); aherland@kth.se (A.H.); 3 AIMES, Department of Neuroscience, Karolinska Institute, 17177 Solna, Sweden; 4 Institute of Systems, Molecular and Integrative Biology, University of Liverpool, Liverpool L69 3BX, UK; K.Ramsbottom@liverpool.ac.uk (K.A.R.); drigden@liverpool.ac.uk (D.J.R.); 5 Computational Biology Facility, Technology Directorate, University of Liverpool, Liverpool L69 3BX, UK

**Keywords:** bisphenols, BPA, BPF, BPS, blood-brain barrier, BBB, BCRP, ABCG2, breast cancer resistance protein

## Abstract

The breast cancer resistance protein (BCRP) is an important efflux transporter in the blood-brain barrier (BBB), protecting the brain from a wide range of substances. In this study, we investigated if BCRP function is affected by bisphenol A (BPA), a high production volume chemical used in common consumer products, as well as by bisphenol F (BPF) and bisphenol S (BPS), which are used to substitute BPA. We employed a transwell-based in vitro cell model of iPSC-derived brain microvascular endothelial cells, where BCRP function was assessed by measuring the intracellular accumulation of its substrate Hoechst 33342. Additionally, we used in silico modelling to predict if the bisphenols could directly interact with BCRP. Our results showed that BPA significantly inhibits the transport function of BCRP. Additionally, BPA was predicted to bind to the cavity that is targeted by known BCRP inhibitors. Taken together, our findings demonstrate that BPA inhibits BCRP function in vitro, probably by direct interaction with the transporter. This effect might contribute to BPA’s known impact on neurodevelopment.

## 1. Introduction

The blood-brain barrier (BBB) is the interface between systemic blood circulation and the central nervous system (CNS). This tight barrier consists of endothelial cells of the capillary walls in a complex network with astrocytes and pericytes. By controlling the flux of nutrients, metabolites, and drugs between the blood and CNS, the BBB is the gatekeeper of brain functionality. Impairment of BBB function contributes to the pathology of neurological conditions, including multiple sclerosis, stroke, epilepsy, and Alzheimer’s disease [1,2,3,4,5]. In addition, BBB integrity has been implicated in playing a role in neurodevelopmental disorders, such as autism spectrum disorder [6].

Due to tight junctions between the endothelial cells, the majority of molecules cannot diffuse passively through the BBB but are actively transported by the BBB’s many different transporters. One group of BBB transporters is comprised of the ATP-binding cassette (ABC) transporters [7,8,9], including the breast cancer resistance protein (BCRP; coded by the gene *ABCG2*). The BCRP is an efflux transporter that can actively export a range of endogenous and exogenous substrates from the brain to the blood, thereby facilitating the maintenance of brain homeostasis, as well as protecting the brain against xenobiotics [7,8,9,10]. The BCRP transporter is not specific for the BBB but is highly expressed in barrier cells in, e.g., the colon, small intestine, placenta, and liver [11,12].

Genetic variants of the BCRP have been associated with different treatment-resistant cancers, gout, and Alzheimer’s disease [13]. Alzheimer’s disease is the most common neurodegenerative disorder worldwide, where affected individuals suffer from cognitive deficits, as well as behavioral changes [14]. The disease is thought to start with overproduction and impaired clearance of β-amyloid (Aβ) peptides, resulting in extracellular Aβ plaque deposition in the brain, neuronal toxicity, and brain atrophy [14]. Dysfunction of ABC transporters has been implicated to play a role in the pathogenesis of Alzheimer’s disease [15]. For example, the BCRP seems to play an essential role in preventing blood-borne Aβ from entering the brain, as *Abcg2*-knock out mice intravenously injected with Aβ display significantly more Aβ accumulation in their brains compared to WT mice [16,17]. Furthermore, in vitro studies have shown that the BCRP can transport Aβ out of brain endothelial cells [18,19].

Bisphenols are a group of endocrine-disrupting chemicals found in plastics, food, thermal receipt papers, dust, and personal care products [20]. The most produced bisphenol is bisphenol A (BPA), but the use of structural analogues, such as bisphenol F (BPF) and bisphenol S (BPS), is increasing to substitute BPA in different products. The ubiquitous use of these chemicals can be reflected by their detection in urine [21,22,23,24,25] and brain [26] of most adults, as well as in umbilical cord blood [27]. Exposure to bisphenols has been shown to affect, for example, neurodevelopment in many animal studies [28], and epidemiological studies are supporting these experimental findings. For example, prenatal BPA exposure has been repeatedly associated with changes in child neurobehavior [29], and prenatal BPF exposure has been associated with lower cognition in childhood [22]. In addition, rats exposed to BPA in utero displayed disrupted expression of genes involved in Alzheimer’s disease [30], and rat brain capillaries exposed to BPA ex vivo showed a decreased BCRP function [31]. However, whether BPA affects human BCRP function in the BBB is not known, nor have the BPA analogues BPS and BPF been studied in this context. Therefore, the aim of this study was to assess if BPA, BPF, and BPS affect BCRP function in the human BBB, using both a human in vitro BBB model and in silico modelling.

## 2. Results

### 2.1. Characterization of the In Vitro BBB Model

To investigate the effect of BPA, BPS, and BPF on BCRP function in the BBB, we used a human in vitro BBB model of iPSC-derived human-induced brain microvascular endothelial-like cells (BMECs) grown on transwell^®^ permeable supports. The advantage of this model is the generation of barrier-forming cells that have an in vivo-like resistance, termed transepithelial electrical resistance (TEER) [32,33], as well as expression of proteins characteristic for human BBB, e.g., tight junction proteins and BBB transporters such as the BCRP [34,35,36,37,38].

First, to ensure that any observed effects were not due to cytotoxicity of the bisphenols, we measured lactate dehydrogenase (LDH) release by the cells upon exposure to 500 nM bisphenols for 48 h. This treatment did not induce cytotoxicity in our model (Figure S1).

**Figure 1:**
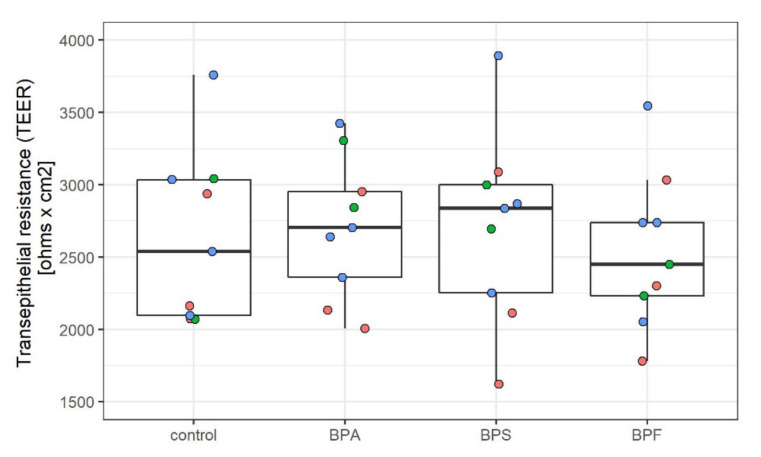
Effect of bisphenols on barrier integrity. Each dot represents the average transepithelial resistance (TEER) of three transwells within one experiment, and the overlying box plots depict median value and the interquartile range (IQR) plus whiskers up to the lowest/highest value <1.5 × IQR. Red = BMECs derived with protocol 1, i.e., hypoxia 10 days protocol (*n* = 3), Green *=* BMECs derived with protocol 2, i.e., normoxia 8 days protocol (*n =* 2), Blue = BMECs derived with protocol 3, i.e., protocol 2, but frozen day 6 and exposed only in transwells (48 h) (*n =* 4). The Kruskal–Wallis test showed no significant differences between the treatment groups.

Subsequently, the general effect of the bisphenols on barrier integrity was investigated. Different protocols to derive the BMECs were used during the study (see Section 4.1 Material and Method), and, as seen in Figure 1, exposure to 500 nM bisphenols did not significantly affect barrier integrity, measured as TEER, using any of the differentiating protocols. More specifically, 500 nM BPA, BPS, and BPF exposure did not affect the barrier integrity, neither when exposure occurred throughout the whole differentiation process (8–10 days exposure; Protocol 1 and 2) nor when exposure only occurred for 48 h (Protocol 3).

### 2.2. Effect of Bisphenol Exposure on BCRP Function

To investigate if the bisphenols could specifically inhibit the efflux transporter BCRP in this model, intracellular accumulation of the BCRP substrate Hoechst 33342 was measured. An increase in intracellular concentration indicates blockage of the BCRP transporter. Cells were exposed to bisphenols either for the whole differentiation protocol (8–10 days) or for the last 2 h prior to analysis to distinguish between effects during the establishment of the barrier and on the already formed barrier.

**Figure 2:**
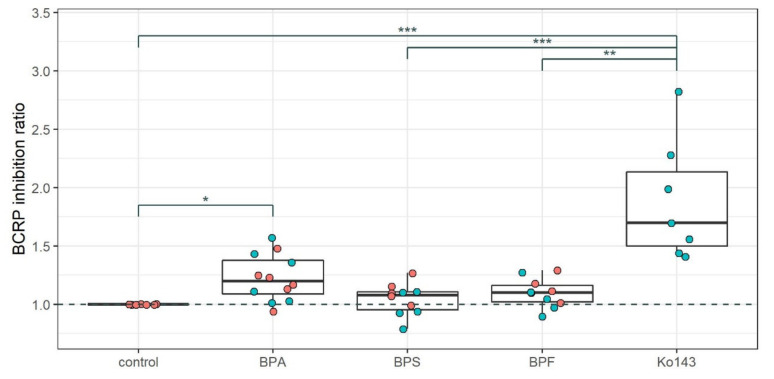
BCRP inhibition by bisphenols in the BBB model. BCRP inhibition = Intracellular Hoechst 33342 accumulation relative to control, where an increased signal indicates less transport of this fluorescent BCRP substrate out of the cells. Each dot represents relative Hoechst accumulation, and the overlying box plots depict median value and the interquartile range (IQR) plus whiskers up to the lowest/highest value < 1.5 × IQR. Pink = 8–10 days exposure; blue = 2 h exposure. Data stems from 7 different experiments, all using protocol 1 or 2. Not all experiments contained both treatment times or all treatments. *n*(Ko143) = 7, *n*(BPA) = 12, *n*(BPS) = 10, *n*(BPF) = 10. Ko143 = known BCRP inhibitor. * = *p* < 0.05, ** = *p* < 0.01, *** = *p* < 0.001 indicate statistical significance, obtained with the Kruskal–Wallis test and Dunn’s post-hoc test using FDR correction for multiple testing.

There was no significant difference in BCRP inhibition between 8–10 days treatment and 2 h treatment. Therefore, we combined the 2 h exposure and 8–10 days exposure settings into one analysis. In this analysis, BPA exposure led to a significant increase in intracellular accumulation of the BCRP substrate Hoechst 33342, similar to that induced by the known BCRP blocker Ko143 (Figure 2). No significant effect of BPF and BPS was observed in this assay.

The BCRP protein is coded by the *ABCG2* gene, and exposure to BPA, BPS, or BPF for 48 h or 8 days did not significantly affect *ABCG2* expression, assessed by qPCR (Figure S2).

### 2.3. In Silico Modelling of Bisphenol-BCRP Binding

The fact that 2 h and 8–10 days bisphenol exposure gave similar BCRP blocking effects and that *ABCG2* expression was not altered by bisphenol exposure suggests that the observed BPA-induced intracellular Hoechst accumulation could be due to a direct physical block of the BCRP transporter. To further investigate this hypothesis, we performed an in silico docking analysis using two different docking programs: AutoDockFR and ROSIE.

#### 2.3.1. Validation of the Method

To validate the accuracy of the two docking programs, we compared the distance between the predicted and known binding positions for the BCRP inhibitor MZ29. AutoDockFR predicted the known binding conformation most accurately, with the lowest root-mean-square deviation (RMSD; 2.5 Å) (Figure S3). Both AutoDockFR and ROSIE accurately predicted MZ29 to bind to cavity 1, which was the expected binding site [39].

#### 2.3.2. Predicted Docking of the Bisphenols to the BCRP Transporter

**Figure 3:**
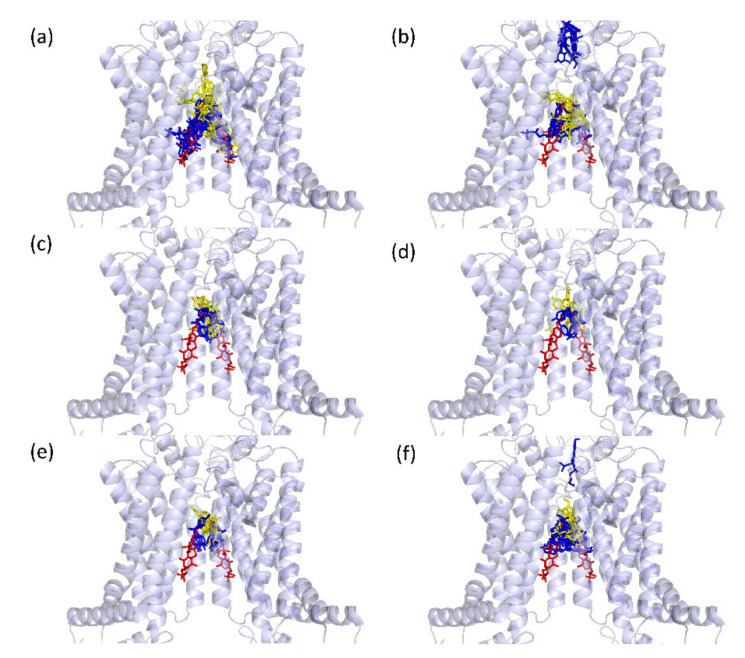
All 10 predicted docking positions for (**a**) MZ29 (known BCRP inhibitor), (**b**) Ko143 (known BCRP inhibitor), (**c**) BPA, (**d**) BPF, (**e**) BPS, and (**f**) MZ40 (known poor BCRP inhibitor) in the protein structure of inhibitor-bound BCRP (Protein Data Bank ID: 6 ETI). Red = MZ29 previously reported position. Blue = predicted docking sites by AutoDockFR. Yellow = predicted docking sites by ROSIE.

When investigating docking positions of the different bisphenols, AutoDockFR and ROSIE gave similar results. All 10 docking positions predicted by the two programs are shown in Figure 3, and the lowest-scoring docking positions are shown in Figure S4. These figures illustrate that both programs predict all three bisphenols to dock in cavity 1, indicating that they have the ability to block this transporter. AutoDockFR, but not ROSIE, predicted Ko143 and MZ40 binding both in Cavity 1 and Cavity 2. ROSIE predicted one pose for BPS and one pose for BPF to be neither in Cavity 1 nor 2. However, these were predicted to bind alongside Cavity 1.

**Figure 4:**
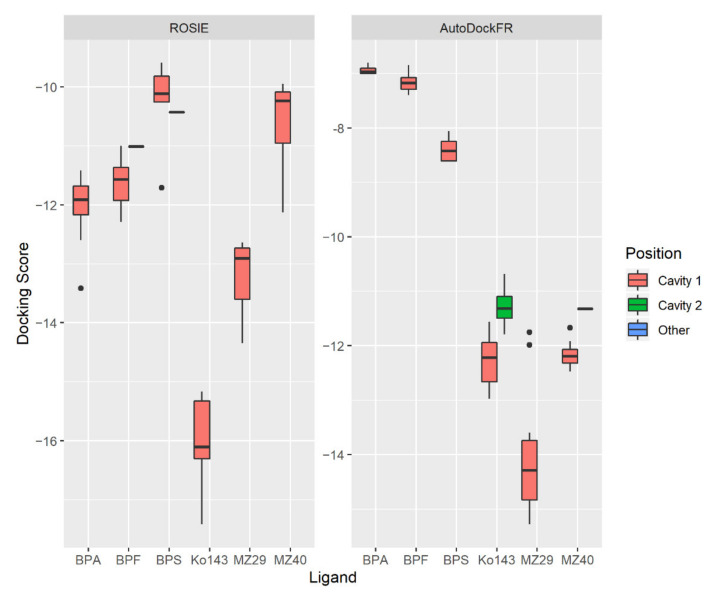
Docking score values for the binding of ligands to the BCRP, obtained by ROSIE and AutoDockFR. The lower the score, the better the predicted binding between the ligand and the BCRP. Ten docking scores were predicted for each compound, and the boxplots show median value and the interquartile range (IQR) plus whiskers up to the lowest/highest value < 1.5 × IQR. Dots show outlier values. ROSIE predicted one pose for BPF and BPS to be outside Cavity 1 and 2 (entitled “Other” in the graph). AutoDockFR predicted one pose in Cavity 2 for MZ40. As there was only one occurrence for each of these poses, these docking scores are shown as horizontal black lines.

When comparing the docking scores of the two programs, ROSIE showed the most favorable binding for Ko143 and MZ29 (known good inhibitors), with less favorable binding for MZ40 (known poor inhibitor), as would be expected [39]. BPA showed the most favorable binding of the bisphenols (Figure 4), which is in line with our experimental findings (Figure 2). AutoDockFR could not distinguish between the Ko143 and MZ40 docking ability (Figure 4), which points to a weakness of this prediction. With AutoDockFR, the bisphenols showed much weaker docking scores than the other substances, with BPS having the most favorable docking score of the bisphenols.

As a next step, binding affinities for the different ligands were predicted. Based on the inability of AutoDockFR to distinguish between Ko143 and MZ40 docking to BCRP, binding affinities were only calculated from the ROSIE docking scores. The CSM-lig program predicted BPA to have the strongest binding affinity of the three bisphenols, although it was lower than the known inhibitors (Figure S5a). The Prodigy-lig program also predicted BPA to have higher BCRP binding affinity than BPS and BPF, but lower affinity than MZ29, Ko143, and MZ40 (Figure S5b).

In conclusion, BPA is predicted to bind to cavity 1 of the BCRP protein, albeit less strong than the known BCRP inhibitor Ko143. This binding could underlie the blocking effect of BPA observed experimentally.

## 3. Discussion

In this study, we investigated the effect of BPA, BPS, and BPF on BCRP function and found that 500 nM BPA inhibits BCRP efflux transportation in a human BBB in vitro model. Although BPF and BPS showed the same trend as BPA, neither BPF nor BPS significantly inhibited BCRP function in our model. The results for BPA is in line with a study by Nickel and Mahringer [31], where 6 h treatment of BPA resulted in inhibited BCRP-mediated transport of the fluorescent BCRP substrate BODIPY-Prazosin from the medium (simulating the brain) into rat brain capillary lumen. More specifically, 500 nM BPA resulted in a decrease from 100% to 86% luminal accumulation of this substrate [31]. Both this and our study could not detect any significant difference in BCRP expression after 500 nM BPA exposure, indicating a physical block of the transporter. We further studied this assumption by modelling bisphenol docking to the BCRP protein, where we observed that the bisphenols were predicted to bind to the same cavity as known inhibitors. The two programs, ROSIE and AutoDockFR, showed slightly different results, which was expected due to the differences in prediction algorithms between the software.

The BPA concentration used in this study (500 nM) was approximately 15–40 times higher than the mean BPA concentration in human serum/blood [40,41,42]. However, it was less than double the highest concentration measured in the blood of pregnant women (*n =* 300; range ND-66.5 ng/mL) [41], indicating that the results obtained are of human relevance for the highest exposed individuals. This study did not assess other concentrations than 500 nM. It will be the task of future studies to address BPA’s IC50 value for BCRP blocking.

Furthermore, BPA is not the only environmental chemical with the ability to inhibit BCRP function. For example, low concentrations of tetrabromobisphenol A (TBBPA; a commonly used brominated flame retardant) and GenX (CAS no. 62037-80-3; used as a replacement for perfluorooctanoic acid (PFOA) in consumer products, such as frying pans) have been shown to inhibit BCRP-mediated transport in rat brain capillaries [43,44]. In addition, many commonly used therapeutic drugs inhibit BCRP transporter activity [18]. Thus, as real-life exposure entails a combination of BPA and other chemicals affecting BCRP function, our findings are relevant for the general population.

The BCRP in the BBB plays an important role in protecting the brain from xenobiotic substances, including environmental chemicals, such as BPA itself [45,46]. Hence, blocking BCRP-mediated efflux is likely to result in higher chemical exposure of the brain, both pre- and postnatally. Thus, the effect of BPA described in this study could contribute to the suspected and known neurodevelopmental adversities associated with exposures to environmental chemicals [47,48,49,50]. In addition, BCRP function is associated with Alzheimer’s disease [13], likely by protecting the brain from blood-borne Aβ [16,17]. Therefore, a block of BCRP function by BPA, alone or in combination with other chemicals, may lead to an increased risk for this neurodegenerative disease. Furthermore, BCRP is expressed in other barriers, such as the intestinal and placental barriers [11], where it also plays important roles. Thus, blocking of the BCRP by BPA could also impact on other tissues than the brain.

In conclusion, our study shows that BPA inhibits BCRP function in a human cell model, probably via direct blocking of this transporter, which is likely contributing to BPA’s impact on human health.

## 4. Materials and Methods

### 4.1. Cells, Chemicals, and Protocols

A human in vitro cell model of the BBB with three slightly different protocols was used (Figure S6), all based on differentiation of the iPSC line iPS(IMR90)-4 (WiCell, Madison, WI, USA) into brain microvascular endothelial-like cells (BMECs). This model was chosen because it gives a very high barrier strength, higher than other models [51], with close to in vivo transepithelial resistance (TEER), and because these BMECs express BBB endothelial cell markers, e.g., tight junction proteins and ABC transporters [34,35,36,37,38], and BCRP function has been confirmed in these cells [52,53]. In addition, this model provides the possibility to expose the cells during development. Regardless of the protocol used in this study, during the last 48 h prior to analyses, the cells were grown on transwell^®^ membranes (24-well plates with 0.4 μm pore polyester cell culture membrane inserts; Corning, NY, USA) and pre-coated with collagen IV from human placenta (Sigma Aldrich, St. Louis, MO, USA) and bovine plasma Fibronectin (Sigma-Aldrich, Inc., St. Louis, MO).

Stocks of BPA (Sigma-Aldrich; Cat no 42088, St. Louis, MO), BPS (Sigma-Aldrich; Cat no 43034, St. Louis, MO) and BPF (Sigma-Aldrich; Cat no 51453, St. Louis, MO) were made in DMSO (Sigma-Aldrich, St. Louis, MO), and cells were exposed to either 500 nM bisphenol or an equal volume of DMSO only. All treatments were performed in three technical replicates per experiment.

#### 4.1.1. Cell Culture Protocol 1 (Hypoxia; 10 Days Protocol)

Protocol 1 was adapted from [34]. iPS(IMR90)-4 cells were seeded in Matrigel (Corning) coated 6-well plates three days before differentiation start at a cell density of 10–12 × 10^4^ cells/cm^2^ in mTeSR medium (Stemcell technologies, Vancouver, Canada), supplemented with 10 µM Y-27632 (Tocris, Bristol, UK). On day 0, differentiation was initiated by changing medium to DMEM/F12 + glutamax (Gibco™; Thermo Fisher Scientific, Inc., Waltham, MA, USA), supplemented with 20% KnockOut Serum Replacement (Gibco, Waltham, MA, USA), 1x MEM Non-Essential Amino Acids Solution (Gibco, Waltham, MA, USA), and 50 µM 2-Mercaptoethanol (Gibco, Waltham, MA, USA). Cultures were incubated at 37 °C with 5% CO_2_ and 5% O_2_ (hypoxia condition). On day six, the culture medium was changed to Human Endothelial-SFM (Gibco, Waltham, MA, USA), with 1% bovine platelet-poor plasma-derived serum (Alfa Aesar™, Thermo Fisher (Kandel) GmbH, Kandel, Germany) (referred to as h-endo medium 1), supplemented with 10 µM retinoic acid (RA; Sigma Aldrich, St. Louis, MO) and 20 ng/mL bFGF (R&D systems, Minneapolis, MN, USA).

On day eight, cells were detached using TrypLE Select Enzyme (Gibco, Waltham, MA, USA) and seeded into transwells at a seeding density of 10^6^ cells/cm^2^ in h-endo medium 1. After 24 h, the medium was changed to fresh h-endo medium 1 but without RA or bFGF, and the plates were moved from hypoxia to normoxia (37 °C with 5% CO_2_ and 20–21% O_2_). After yet another 24 h, on day 10, different analyses were performed.

Bisphenols or DMSO were introduced to the medium from day 0, i.e., exposure during the whole differentiation process (10 days). In addition, for the BCRP analyses, bisphenols were introduced to control (DMSO) cells 2 h prior to the BCRP assay. The medium, including bisphenols/DMSO, was changed every day except on day 7.

#### 4.1.2. Cell Culture Protocol 2 (Normoxia; 8 Days Protocol)

Protocol 2 was adapted from [35]. iPS(IMR90)-4 cells were seeded in Matrigel-coated 6-well plates one day before differentiation start at a cell density of 1.6 × 10^4^ cells/cm^2^ in mTeSR medium supplemented with 10 µM Y-27632. On day 0, differentiation was initiated by changing the medium to Essential 6 Medium (Gibco, Waltham, MA, USA). On day 4, the medium was changed to Human Endothelial-SFM, with 1× B-27 Supplement (Gibco, Waltham, MA, USA) (referred to as h-endo medium 2), supplemented with 10 µM RA and 20 ng/mL bFGF.

On day six, cells were detached using TrypLE Select Enzyme and seeded into transwells at a seeding density of 10^6^ cells/cm^2^ in h-endo medium 2. After 24 h, the medium was changed to fresh h-endo medium 2 but without RA or bFGF. The plates were incubated for another 24 h before the analyses.

Bisphenols or DMSO were introduced to the medium from day 0, i.e., exposure during the whole differentiation process (8 days). In addition, for the BCRP analyses, bisphenols were introduced to control (DMSO) cells 2 h prior to the BCRP assay. The medium, including bisphenols/DMSO, was changed every day except on day 5.

#### 4.1.3. Cell Culture Protocol 3 (Normoxia with Freezing at Day 6; 2 Days Protocol)

To shorten the experimental protocol and potentially decrease variability between experiments, protocol 3 was introduced. Here, untreated cells from Protocol 2 day 6 were cryopreserved (in 10% DMSO, 40% FBS, 50% h-endo medium 2) after detachment from the 6-well plates [36]. In protocol 3, the cryopreserved cells were thawed and seeded in transwells, as in protocol 2, with the addition of 10 µM Y-27632 to the medium. As in protocol 2, the medium was changed to fresh h-endo medium 2 after 24 h and the plates were incubated for another 24 h before the analyses.

Bisphenols or DMSO were added when the cells were seeded into transwells, i.e., after 48 h exposure.

### 4.2. Analyses of the BBB Cell Model

#### 4.2.1. Cytotoxicity

At the assay day, the medium from the top compartment was analyzed using CytoTox 96^®^ Non-Radioactive Cytotoxicity Assay (Promega, Madison, WI, USA) according to the manufacturer’s instructions. This assay measures release of LDH, an indicator of cytotoxicity. LDH release was analyzed in triplicate transwells of three separate experiments, all using protocol 3.

#### 4.2.2. TEER

On the assay day (48 h after seeding into transwells), barrier integrity was assessed by measuring the electrical resistance (Ω) of the barrier using an EVOM2 instrument with STX2 electrodes (World Precision Instruments, Sarasota, FL, USA). Background resistance of a “blank” transwell was subtracted from all samples, after which the measured resistance was multiplied by the surface area of the transwell (0.33 cm^2^) to obtain the transepithelial electrical resistance (TEER) in Ω*cm^2^.

#### 4.2.3. BCRP Function

On the assay day (48 h after seeding into transwells), BCRP function was evaluated by measuring the intracellular accumulation of the BCRP substrate Hoechst 33342 (Invitrogen™, Thermo Fisher Scientific, Inc, Waltham, MA, USA). In brief, the cells were incubated with 20 µM Hoechst in medium for 1 h at 37 °C, with 5% CO_2_, after which the cells were washed three times with Dulbecco’s phosphate-buffered saline (DPBS; Gibco, Waltham, MA, USA) and lysed with 100 uL cold radioimmunoprecipitation assay buffer (RIPA; Thermo Scientific™, Thermo Fisher Scientific, Inc, Waltham, MA, USA). Homogenized lysate (80 µL) was transferred to black ELISA plates (ThermoFisher Scientific) and the fluorescence intensity was measured (350 nm ex/460 nm em) using a TECAN Infinite reader (TECAN, Männedorf, Switzerland).

As a positive control, cells were exposed to 100 nM Ko143 hydrate (a known BCRP inhibitor; Sigma–Aldrich, St. Louis, MO) for 2 h. For this, Ko143 was added to DMSO control cells 1 h prior to and during Hoechst incubation.

The average Hoechst signal of the treatment was divided by the average signal of the control and presented as a ratio. A higher ratio means less Hoechst transport out of the cells and thus, blockage of BCRP transport.

#### 4.2.4. Gene Expression

On the assay day (48 h after seeding into transwells), cells were washed with DPBS and lysed using RLT plus buffer (Qiagen, Hilden, Germany), supplemented with 2-Mercaptoethanol (Sigma-Aldrich, St. Louis, MO) in the transwells. The lysates were frozen and kept at −80 °C until RNA was extracted using the AllPrep^®^ DNA/RNA Micro kit (Qiagen), according to protocol.

To analyze BCRP (*ABCG2*) gene expression using qPCR, extracted RNA (100–200 ng depending on the experiment) from three different experiments, using protocol 2 or 3, were converted to cDNA using iScript™ gDNA Clear cDNA Synthesis Kit (Bio-Rad, Hercules, CA, USA). Expression levels were analyzed by mixing cDNA, primers, and qPCR SsoAdvanced Universal SYBR Green Supermix (Bio-Rad) in a total volume of 10 µL, according to the manufacturer’s instructions. To analyze reference genes, 0.3 µM forward, and reverse primers for Beta-2-microglobulin (B2M), Hypoxanthine phosphoribosyl-transferase 1 (HPRT1), and TATA box binding protein (TBP) were used; primer sequences are presented in Table S1. For ABCG2, PrimePCR™ SYBR^®^ Green Assay (Bio-Rad) was used. Amplification was measured in a C1000 Touch™ Thermal Cycler with a 384-Well Reaction Module (Bio-Rad) using a 2 min incubation at 95 °C followed by 40 cycles of 5 s at 95 °C and 30 s at 60 °C. All assays had similar PCR efficiency, and the reference genes were not affected by bisphenol treatment. Samples from three independent experiments were analyzed, where cDNA from at least two transwells per treatment was available. cDNA from each transwell was analyzed in triplicate wells in the 384-well PCR plate. Relative expression was calculated using the 2^-∆∆Ct^ method [54,55], where the geometric mean of the three reference genes’ C_T_ values was used to calculate the ∆C_T_ to the *ABCG2* C_T_ value in all samples. The average ∆C_T_ of the three DMSO controls was used to calculate the ∆∆C_T_ to all samples, and the average ∆∆C_T_ per treatment within one experiment was used. The 2^-∆∆CT^ values were calculated, which were then Log2 transformed (LogFC).

#### 4.2.5. Statistics

To compare TEER values, BCRP blockage, BPRP expression (qPCR), LDH release between the treatment groups (DMSO, BPA, BPS, BPF), and the Kruskal–Wallis test were applied. If this test was significant, Dunn’s test of multiple comparisons was applied, where *p*-values were adjusted with the Benjamini–Hochberg method. When comparing BCRP inhibition following 2 h and 8–10 days treatment, the paired samples Wilcoxon test was used. For all tests, a *p* value < 0.05 was regarded as significant. Graphs and statistics for the in vitro experiments were made in RStudio, using R version 4.0.2.

### 4.3. Modelling of Bisphenol Binding to BCRP

#### 4.3.1. Docking Programs Used

Two freely available and commonly used docking programs were used for predicting the binding to BCRP: Rosetta Online Server that Includes Everyone (ROSIE) (https://rosie.graylab.jhu.edu/ligand_docking, accessed in July 2019) [56,57] and AutoDockFR v1.2 (Center for Computational Structural Biology, La Jolla, CA, USA). The protein structure of inhibitor-bound ABCG2, ID: 6ETI [39], was obtained through the Protein Data Bank (PDB) and used as the target protein in the molecular docking.

Using ROSIE, both chain A and B were used. The starting coordinates were given as cavity one [39], where MZ29 (PDB compound ID: BWQ) was seen bound in 6ETI. The ligand files were converted to SDF format and ligand conformers were generated.

Using AutoDockFR, both chain A and B were used. The target and ligand files were converted to PDBQT files using AutoDock Tools [58]. AutoGridFR v1.2 (Center for Computational Structural Biology, La Jolla, CA, USA) [59] was used to generate affinity maps, searching the space surrounding cavities one and two. As AutoDockFR only gives the binding pose for the top binding solution, AutoDockFR was run in batches to obtain multiple binding poses. The top pose was therefore given for a selection of 10 runs as opposed to the top poses for one run seen using ROSIE.

#### 4.3.2. Prediction of Binding Positions

First, we wanted to investigate if these docking programs could accurately predict the binding of known BCRP inhibitors. For this, we used the known inhibitors MZ29 and Ko143, as well as the poor inhibitor MZ40 [39]. MZ29 (BWQ) structure was extracted directly from the 6ETI PDB file, Ko143 was obtained from the ZINC database (ZINC ID: 35636075), MZ40 was sketched from the Ko143 structure using Molinspiration (www.molinspiration.com, accessed on 28 June 2019), and the topology was predicted using the PRODRG server [60].

The bound MZ29 was removed from the BCRP (6ETI), and the docking software was used to re-dock the MZ29 inhibitor back into the protein. This allowed us to determine how well the two programs were able to predict the correct binding position of the MZ29 inhibitor. In order to determine how closely the predictions recreated the known docking position, root-mean-square deviations (RMSDs) were calculated from the original structure. As the 6ETI structure shows two MZ29 molecules bound, RMSDs were calculated from each of the two molecules, and the lowest RMSD was reported.

In addition to the known inhibitors, docking poses were also predicted for BPA, BPF, and BPS in the same way as the known inhibitors. The bisphenol structures were obtained from the ZINC database; BPA (ZINC ID: 56434), BPS (ZINC ID: 56964), and BPF (ZINC ID: 136152).

#### 4.3.3. Predicting Binding Affinities for the Bisphenols

Binding affinities were predicted for the compounds using the CSM-lig [61] and Prodigy-lig [62] servers. Both of these programs take the protein-ligand complex and calculate a predicted binding affinity based on structural properties, such as residue interactions. For CSM-lig, the most favorable positions show higher scores, whereas for Prodigy-lig, the most favorable positions show lower scores.

## Data Availability

The data presented in this study are available on request from the corresponding author. The data are not publicly available due to raw data format.

## Supplementary Materials

The following are available online at https://www.mdpi.com/article/10.3390/ijms22115534/s1, Table S1: Primer sequences, Figure S1: Cytotoxicity induced by 48 h bisphenol exposure, Figure S2: ABCG2 expression, Figure S3: Root-mean-square deviations (RMSDs) to previously reported BWQ binding position, Figure S4: Best predicted docking positions, Figure S5: Predicted BCRP binding affinities, Figure S6: Schematic overview of the three protocols used.
