# Supplementary material for: Bisphenol A Inhibits the Transporter Function of the Blood-Brain Barrier by Directly Interacting with the ABC Transporter Breast Cancer Resistance Protein (BCRP)
Source: Int J Mol Sci. 2021 May 24;22(11):5534. doi: 10.3390/ijms22115534 (PMC8197233; doi:10.3390/ijms22115534)
Supplement: Supplementary file 1 [file ijms-22-05534-s001.zip › ijms-1231802-supplementary.pdf]

Supplementary Materials

Bisphenol A Inhibits the Transporter Function of the Blood-Brain Barrier by Directly Interacting with the ABC Transporter Breast Cancer Resistance Protein (BCRP)

Table S1. Primer sequences for the reference genes used.

Gene	Forward primer (5′→3′)	Reverse primer (5′→3′)
HPRT1	TGACACTGGCAAAACAATGCA	GGTCCTTTTCACCAGCAAGCT
TBP	GAGCCAAGAGTGAAGAACAGTC	GCTCCCCACCATATTCTGAATCT
B2M	TGCTGTCTCCATGTTTGATGTATCT	TCTCTGCTCCCCACCTCTAAGT

Hypoxanthine phosphoribosyl-transferase 1 (HPRT1); TATA box binding protein (TBP); Beta-2-microglobulin (B2M)

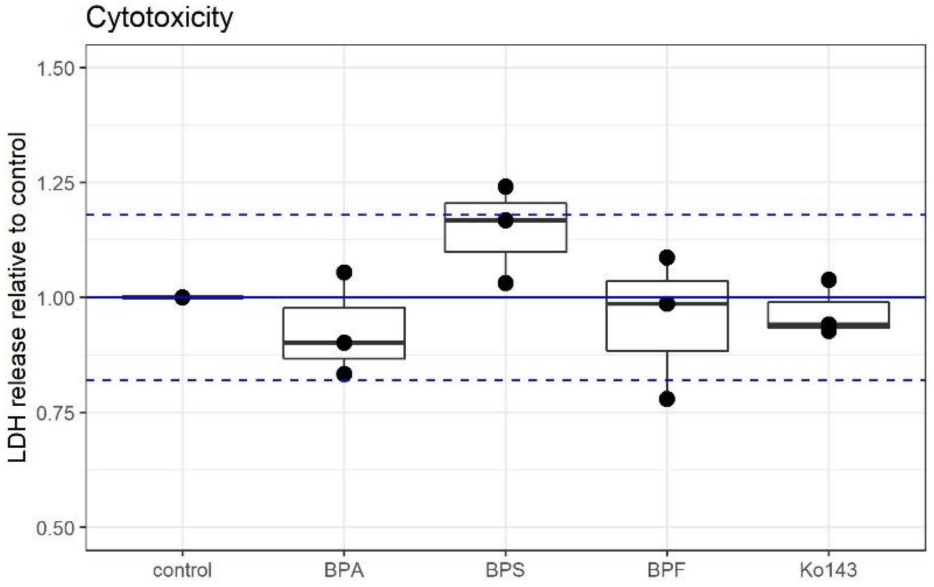

Figure S1. Cytotoxicity induced by 48 h bisphenol exposure. Cytotoxicity was measured as LDH release and expressed as a ratio to the DMSO control. Each dot represents the average relative LDH release of triplicate transwells within one experiment, all obtained using protocol 3. The bisphenol exposure started at seeding in a transwell, the media was changed after 24 h, and, after an additional 24 h, the media was analyzed for LDH release. Absorbance (490 nm) values were obtained for each well and divided by the average of DMSO wells in each experiment. The dotted line indicates the standard deviation of the relative LDH release of the individual DMSO transwells (n = 9) of all three experiments.

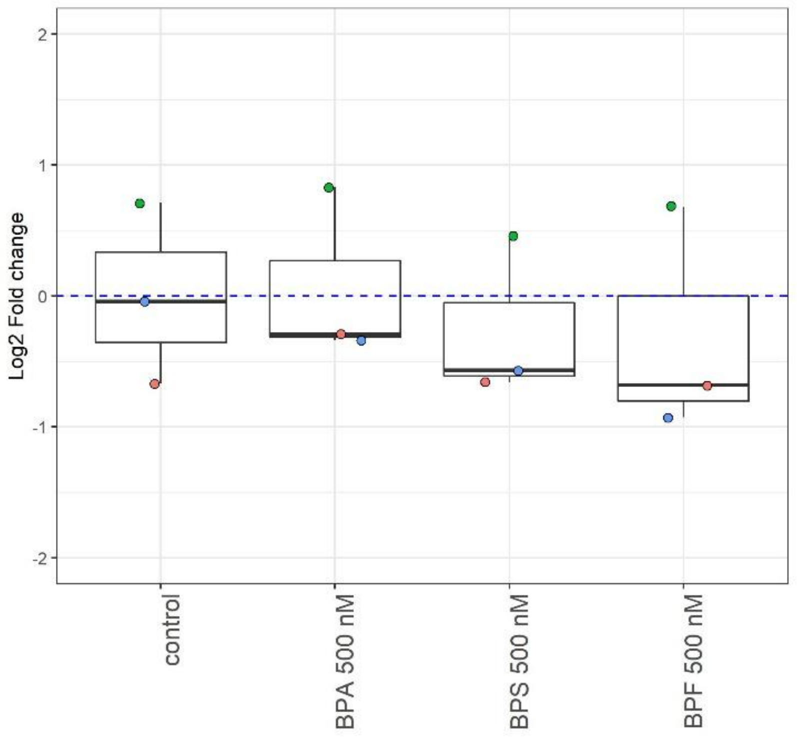

Figure S2. ABCG2 expression, measured by qPCR. Log2 fold change compared to the controls, calculated using the $2^{-\Delta\Delta CT}$ method, with HPRT1, TBP, and B2M as reference genes. Each colour indicates an experiment. n = 3 per treatment group, obtained using protocol 2 (red) and 3 (blue and green).

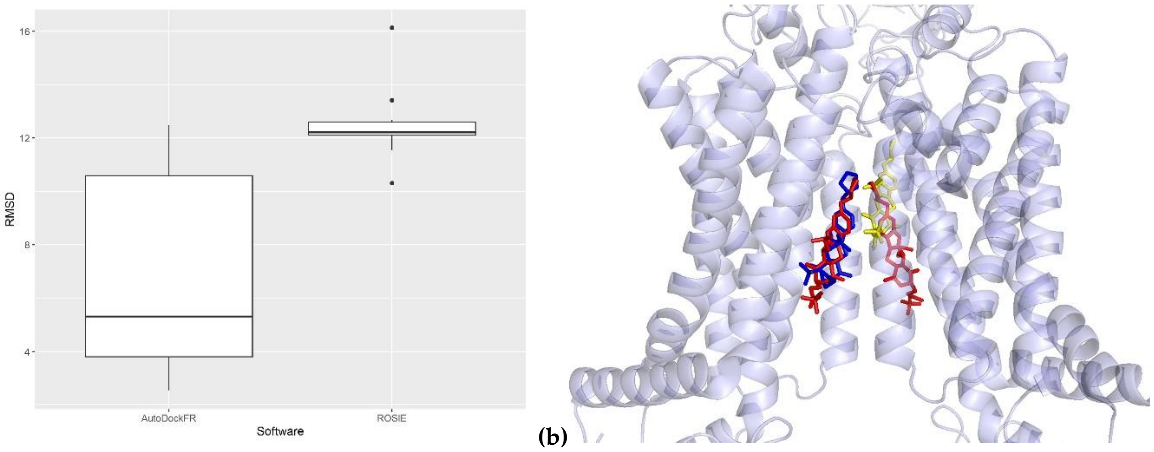

(a) **(b)**

Figure S3. Root-mean-square deviations (RMSDs) of the previously reported MZ29 binding position. (**a**) RMSD for each of the 10 predicted poses for MZ29 using each program; (**b**) poses with the lowest RMSD, measured from MZ29 known positions (shown in red) for AutoDockFR (2.526Å: shown in blue) and ROSIE (10.316Å: shown in yellow).

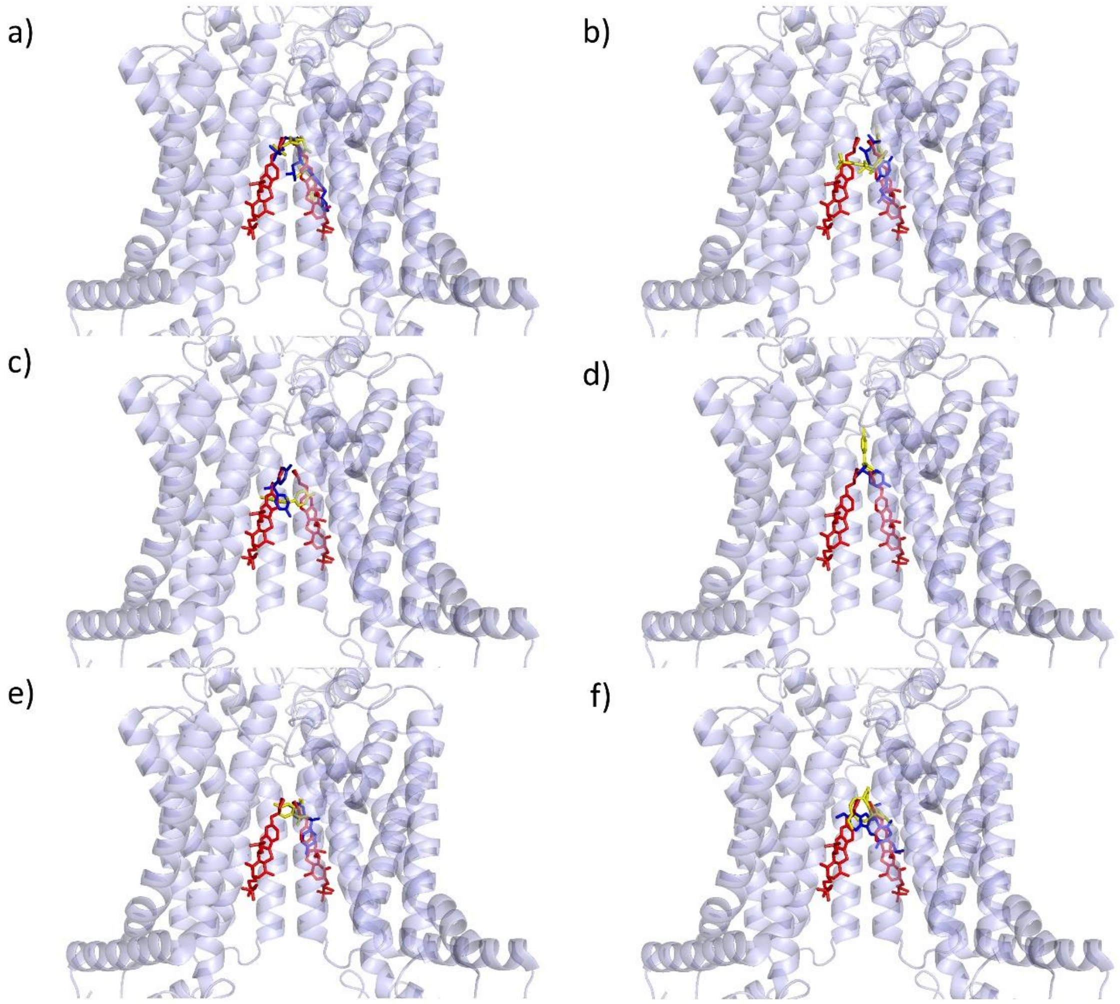

Figure S4. Best predicted docking positions for (**a**) MZ29 (known BCRP inhibitor), (**b**) Ko143 (known BCRP inhibitor), (**c**) BPA, (**d**) BPF, (**e**) BPS, and (**f**) MZ40 (known poor BCRP inhibitor) in the protein structure of inhibitor-bound ABCG2 (Protein Data Bank ID: 6ETI). Red = MZ29's previously reported position. Blue = predicted docking site by AutoDockFR. Yellow = predicted docking site by ROSIE.

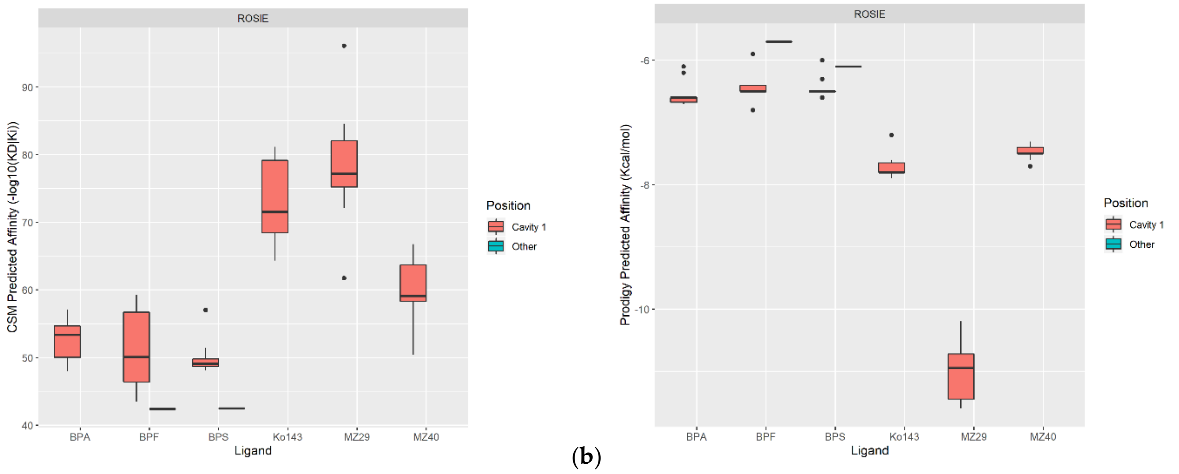

(**a**) (**b**)

Figure S5. Predicted BCRP binding affinities using (**a**) CSM-lig and (**b**) Prodigy-lig. Affinities were predicted on values obtained with the ROSIE software. (**a**) The higher –log10(KD|Ki) score, the better the affinity. (**b**) The lower Kcal/mol score, the better the affinity.

Protocol 1

			**10 d exposure start**										**2h exposure start**
d-3	d-2	d-1	d0	d1	d2	d3	d4	d5	d6	d7	d8	d9	d10
			To hypoxia	hypoxia	hypoxia	hypoxia	hypoxia	hypoxia	hypoxia	hypoxia	hypoxia	To normoxia	
Seeding into 6-well plate (mTesR + ROCKi)	media change mTesR	media change mTesR	Media change to diff media	Media change (diff media)	Media change (diff media)	Media change (diff media)	Media change (diff media)	Media change (diff media)	Media change to h-endo media 1		Seed into **transwells** in h-endo media 1	Media change to h-endo media 2	Assay day

Protocol 2

	**8 d exposure start**								**2h exposure start**
d-1	d0	d1	d2	d3	d4	d5	d6	d7	d8
Seeding into 6-well plate (mTesR + ROCKi)	Media change to E6 media.	Media change (E6)	Media change (E6)	Media change (E6)	Media change to h-endo media 3		Seed into **transwells** in h-endo media 3	Media change to h-endo media 4	Assay day

Protocol 3

							**48 h exposure start**		
d-1	d0	d1	d2	d3	d4	d5	d6	d7	d8
Cells were differentiated with protocol 2 and frozen at d5							Cells thawed in h-endo media 3 + ROCKi, seed into **transwells.**	Media change to h-endo media 4	Assay day

Figure S6. Schematic overview of the three protocols used.
ROCKi = ROCK inhibitor Y-27632 dihydrochloride;
Diff media = DMEM/F12 + glutamax with 20% KnockOut Serum Replacement 1× MEM Non-Essential Amino Acids Solution and 50 µM 2-ME;
h-endo media 1 = Human Endothelial-SFM with 1% bovine platelet-poor plasma derived serum (PDS), 10 µM Retinoic acid (RA) and 20 ng/ml bFGF; h-endo media 2 = Human Endothelial-SFM with 1% PDS;
h-endo media 3 = Human Endothelial-SFM with 1× B27, 10 µM Retinoic acid (RA) and 20 ng/ml bFGF;
h-endo media 4 = Human Endothelial-SFM with 1× B27.
